# The DZHK research platform: maximisation of scientific value by enabling access to health data and biological samples collected in cardiovascular clinical studies

**DOI:** 10.1007/s00392-023-02177-5

**Published:** 2023-03-08

**Authors:** Julia Hoffmann, Sabine Hanß, Monika Kraus, Jens Schaller, Christian Schäfer, Dana Stahl, Stefan D. Anker, Gabriele Anton, Thomas Bahls, Stefan Blankenberg, Arne Blumentritt, Leif-Hendrik Boldt, Steffen Cordes, Steffen Desch, Wolfram Doehner, Marcus Dörr, Frank Edelmann, Ingo Eitel, Matthias Endres, Stefan Engelhardt, Jeanette Erdmann, Katharina Eulenburg, Volkmar Falk, Stephan B. Felix, Derk Frank, Thomas Franke, Norbert Frey, Tim Friede, Lars Geidel, Lisa Germans, Ulrich Grabmaier, Martin Halle, Jörg Hausleiter, Vera Jakobi, Ahmad-Fawad Jebran, Alexander Jobs, Stefan Kääb, Mahir Karakas, Hugo A. Katus, Alexandra Klatt, Christoph Knosalla, Joachim Krebser, Ulf Landmesser, Mahsa Lee, Kristin Lehnert, Stephanie Lesser, Katrin Leyh, Roberto Lorbeer, Stephanie Mach-Kolb, Benjamin Meder, Eike Nagel, Christian H. Nolte, Abdul S. Parwani, Astrid Petersmann, Miriam Puls, Henriette Rau, Maximilian Reiser, Otto Rienhoff, Tabea Scharfe, Mario Schattschneider, Heiko Scheel, Renate B. Schnabel, Andreas Schuster, Boris Schmitt, Tim Seidler, Moritz Seiffert, Barbara-Elisabeth Stähli, Adriane Stas, Thomas J. Stocker, Lukas von Stülpnagel, Holger Thiele, Rolf Wachter, Reza Wakili, Tanja Weis, Kerstin Weitmann, Heinz-Erich Wichmann, Philipp Wild, Tanja Zeller, Wolfgang Hoffmann, Elisabeth Maria Zeisberg, Wolfram-Hubertus Zimmermann, Dagmar Krefting, Titus Kühne, Annette Peters, Gerd Hasenfuß, Steffen Massberg, Thomas Sommer, Stefanie Dimmeler, Thomas Eschenhagen, Matthias Nauck

**Affiliations:** 1 DZHK Main Office, Berlin, Germany; 2 Department of Medical Informatics, University Medical Center Göttingen, Göttingen, Germany; 3 Institute of Epidemiology, Helmholtz Zentrum München, Munich, Germany; 4 Institute of Computer-Assisted Cardiovascular Medicine, Deutsches Herzzentrum der Charité, Augustenburger Platz 1, Berlin, Germany; 5 Institute of Clinical Chemistry and Laboratory Medicine, Matthias Nauck: Spokesman of the Clinical Research Platform of the German Centre for Cardiovascular Research (DZHK), University Medicine Greifswald, Greifswald, Germany; 6 Independent Trusted Third Party of the University Medicine Greifswald, Greifswald, Germany; 7 Department of Internal Medicine and Cardiology, Deutsches Herzzentrum der Charité (DHZC), Berlin, Germany; 8 Institute of Health Center for Regenerative Therapies (BCRT), Berlin, Germany; 9 Institute for Community Medicine, Section Epidemiology of Health Care and Community Health, University Medicine Greifswald, Greifswald, Germany; 10 Department of Cardiology, University Heart and Vascular Center Hamburg-Eppendorf, Hamburg, Germany; 11 Heart Center Leipzig at Leipzig University and Leipzig Heart Science, Leipzig, Germany; 12 Center for Stroke Research Berlin, Charité Universitätsmedizin, Berlin, Germany; 13 Department of Internal Medicine B, University Medicine Greifswald, Greifswald, Germany; 14 Medical Clinic II Cardiology Angiology Intensive Care Medicine, University Heart Center Lübeck, Lübeck, Germany; 15 Klinik Und Hochschulambulanz Für Neurologie, Charité Universitätsmedizin Berlin, Berlin, Germany; 16 ExcellenceCluster NeuroCure, Charité Universitätsmedizin Berlin, Berlin, Germany; 17 German Center for Neurodegenerative Diseases (DZNE), Partner Site Berlin, Berlin, Germany; 18 Institute of Pharmacology and Toxicology, Technical University Munich, Munich, Germany; 19 Institute for Cardiogenetics, University Lübeck, Lübeck, Germany; 20 Department for Cardiothoracic and Vascular Surgery, Deutsches Herzzentrum der Charité (DHZC), Berlin, Germany; 21 Swiss Federal Institute of Technology (ETH) Zurich, Translational Cardiovascular Technologies, Zurich, Switzerland; 22 Department of Internal Medicine III (Cardiology, Angiology, Critical Care), University Hospital Schleswig-Holstein, Campus Kiel, Kiel, Germany; 23 Klinik für Kardiologie, Angiologie und Pneumologie, Universitätsklinikum Heidelberg, Heidelberg, Germany; 24 Medizinische Klinik Und Poliklinik I, Klinikum Der Universität München, LMU München, Munich, Germany; 25 Department of Prevention and Sports Medicine, Faculty of Medicine, University Hospital ‘Klinikum Rechts Der Isar’, Technical University Munich, Munich, Germany; 26 Medizinische Klinik III, Kardiologie, Universitätsklinikum Frankfurt, Frankfurt, Germany; 27 Herz- Und Thorax-Chirurgie, University Medical Center Göttingen, Göttingen, Germany; 28 Department of Intensive Care Medicine, University Medical Center Hamburg-Eppendorf (UKE), Hamburg, Germany; 29 Medical Department of Cardiology, Deutsches Herzzentrum der Charité, Berlin, Germany; 30 Department of Radiology, University Hospital, LMU Munich, Munich, Germany; 31 Institute for Experimental and Translational Cardiovascular Imaging and DZHK Center for Cardiovascular Imaging, Frankfurt, Germany; 32 Department of Neurology With Experimental Neurology, Charite Universitätsmedizin Berlin, Berlin, Germany; 33 Berlin Institute of Health, Berlin, Germany; 34 Institute of Clinical Chemistry and Laboratory Medicine, University Medicine Oldenburg, Oldenburg, Germany; 35 Clinic of Cardiology and Pneumology, University Medical Center Göttingen, Göttingen, Germany; 36 Abteilung Pädiatrie M.S. Kardiologie, Charité Universitätsmedizin Berlin, Berlin, Germany; 37 Klinik Für Kardiologie, Angiologie Und Intensivmedizin, Deutsches Herzzentrum der Charité (DHZC), Berlin, Germany; 38 Klinik Und Poliklinik Für Kardiologie, Universitätsklinikum Leipzig, Leipzig, Germany; 39 Preventive Cardiology and Preventive Medicine, Department of Cardiology, University Medical Center Mainz, Mainz, Germany; 40 Department of Cardiology and Pneumology, University Medical Center of Göttingen, Georg-August University, Göttingen, Germany; 41 Institute of Pharmacology and Toxicology, University Medical Center Göttingen, Göttingen, Germany; 42 Max-Delbrück-Centrum Für Molekulare Medizin (MDC), Berlin, Germany; 43 Institute of Cardiovascular Regeneration, Universitätsklinikum Frankfurt, Frankfurt/Main, Germany; 44 Department of Experimental Pharmacology and Toxicology, University Medical Center Hamburg-Eppendorf (UKE), Hamburg, Germany; 45 German Centre for Cardiovascular Research (DZHK) Partner Site Göttingen, Göttingen, Germany; 46 German Centre for Cardiovascular Research (DZHK) Partner Site Berlin, Berlin, Germany; 47 German Centre for Cardiovascular Research (DZHK) Partner Site Greifswald, Greifswald, Germany; 48 German Centre for Cardiovascular Research (DZHK) Partner Site Hamburg/Kiel/Lübeck, Hamburg/Kiel/Lübeck, Germany; 49 German Centre for Cardiovascular Research (DZHK) Partner Site Munich Heart Alliance, Munich, Germany; 50 German Centre for Cardiovascular Research (DZHK) Partner Site Heidelberg/Mannheim, Heidelberg/Mannheim, Germany; 51 German Centre for Cardiovascular Research (DZHK) Partner Site Rhine/Main, Bad Nauheim/Frankfurt/Mainz, Germany; 52 Charité - Universitätsmedizin Berlin, corporate member of Freie Universität Berlin and Humboldt-Universität zu Berlin, Berlin, Germany; 53 Centre for Population Health Innovation (POINT), University Heart and Vascular Center Hamburg, University Medical Center Hamburg-Eppendorf, Hamburg, Germany; 54 Department Heart and Vascular Diseases, University Hospital Heidelberg, Heidelberg, Germany; 55 Center for Thrombosis and Hemostasis, University Medical Center Mainz, Mainz, Germany; 56 Institute of Molecular Biology (IMB), Mainz, Germany; 57 University Center of Cardiovascular Science, University Heart and Vascular Center Hamburg, Hamburg, Germany; 58 Cluster of Excellence “Multiscale Bioimaging: from Molecular Machines to Networks of Excitable Cells” (MBExC), University of Göttingen, Göttingen, Germany

**Keywords:** Cardiovascular disease, Data and biomaterial collection, German Centre for Cardiovascular Research, Research platform, Standardisation

## Abstract

The German Centre for Cardiovascular Research (DZHK) is one of the German Centres for Health Research and aims to conduct early and guideline-relevant studies to develop new therapies and diagnostics that impact the lives of people with cardiovascular disease. Therefore, DZHK members designed a collaboratively organised and integrated research platform connecting all sites and partners. The overarching objectives of the research platform are the standardisation of prospective data and biological sample collections among all studies and the development of a sustainable centrally standardised storage in compliance with general legal regulations and the FAIR principles. The main elements of the DZHK infrastructure are web-based and central units for data management, LIMS, IDMS, and transfer office, embedded in a framework consisting of the DZHK Use and Access Policy, and the Ethics and Data Protection Concept. This framework is characterised by a modular design allowing a high standardisation across all studies. For studies that require even tighter criteria additional quality levels are defined. In addition, the Public Open Data strategy is an important focus of DZHK. The DZHK operates as one legal entity holding all rights of data and biological sample usage, according to the DZHK Use and Access Policy. All DZHK studies collect a basic set of data and biosamples, accompanied by specific clinical and imaging data and biobanking. The DZHK infrastructure was constructed by scientists with the focus on the needs of scientists conducting clinical studies. Through this, the DZHK enables the interdisciplinary and multiple use of data and biological samples by scientists inside and outside the DZHK. So far, 27 DZHK studies recruited well over 11,200 participants suffering from major cardiovascular disorders such as myocardial infarction or heart failure. Currently, data and samples of five DZHK studies of the DZHK Heart Bank can be applied for.

## Introduction

The German Centre for Cardiovascular Research (DZHK), consisting of seven German partner sites and a total of 28 institutions, was established in 2012 on the initiative of the German Federal Ministry of Education and Research (BMBF) [1]. In addition to the DZHK, there are currently five further German Centres for Health Research (DZG), which are dedicated to translational research on other widespread diseases and another two centres are currently being established [2].

**Fig. 1:**
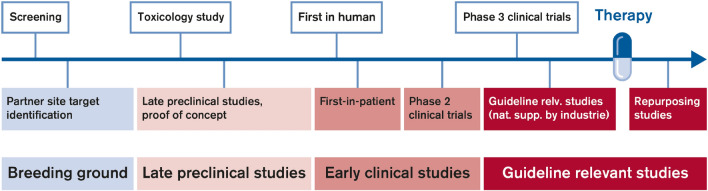
Translational pipeline of the DZHK: from breeding ground to guideline relevant studies

An important part of the DZHK mission is to conduct early and guideline-relevant studies to develop new therapies and diagnostics that impact the lives of people with cardiovascular disease. It brings together outstanding basic and clinical researchers from seven partner sites in Germany (Berlin, Göttingen, Greifswald, Hamburg/Kiel/Lübeck, Heidelberg/Mannheim, Munich, Rhine-Main (Frankfurt/Bad Nauheim/Mainz)). A total of 17 DZHK clinical study units have been implemented at clinics mostly at these partner sites [3]. To achieve its mission, the DZHK aims to rapidly and efficiently transfer results from basic research into clinical practice (Fig. 1). The DZHK fosters co-operation between scientists with the aim of developing synergies and thus accelerating the process of translation. The DZHK primarily invests its budget into the translation process based on a coordinated research strategy and specific funding instruments [4].

Shortly after its initiation, the DZHK founded several working groups, one of them focusing on strategically shaping and designing a state-of-the-art clinical research platform enabling investigator-initiated trials (IITs). Central values of the DZHK are transparency and participation of scientists and clinicians from all partner sites. To meet these expectations, especially in view of clinical studies, the DZHK members designed a collaboratively organised and integrated research platform connecting all sites and partners.

**Fig. 2:**
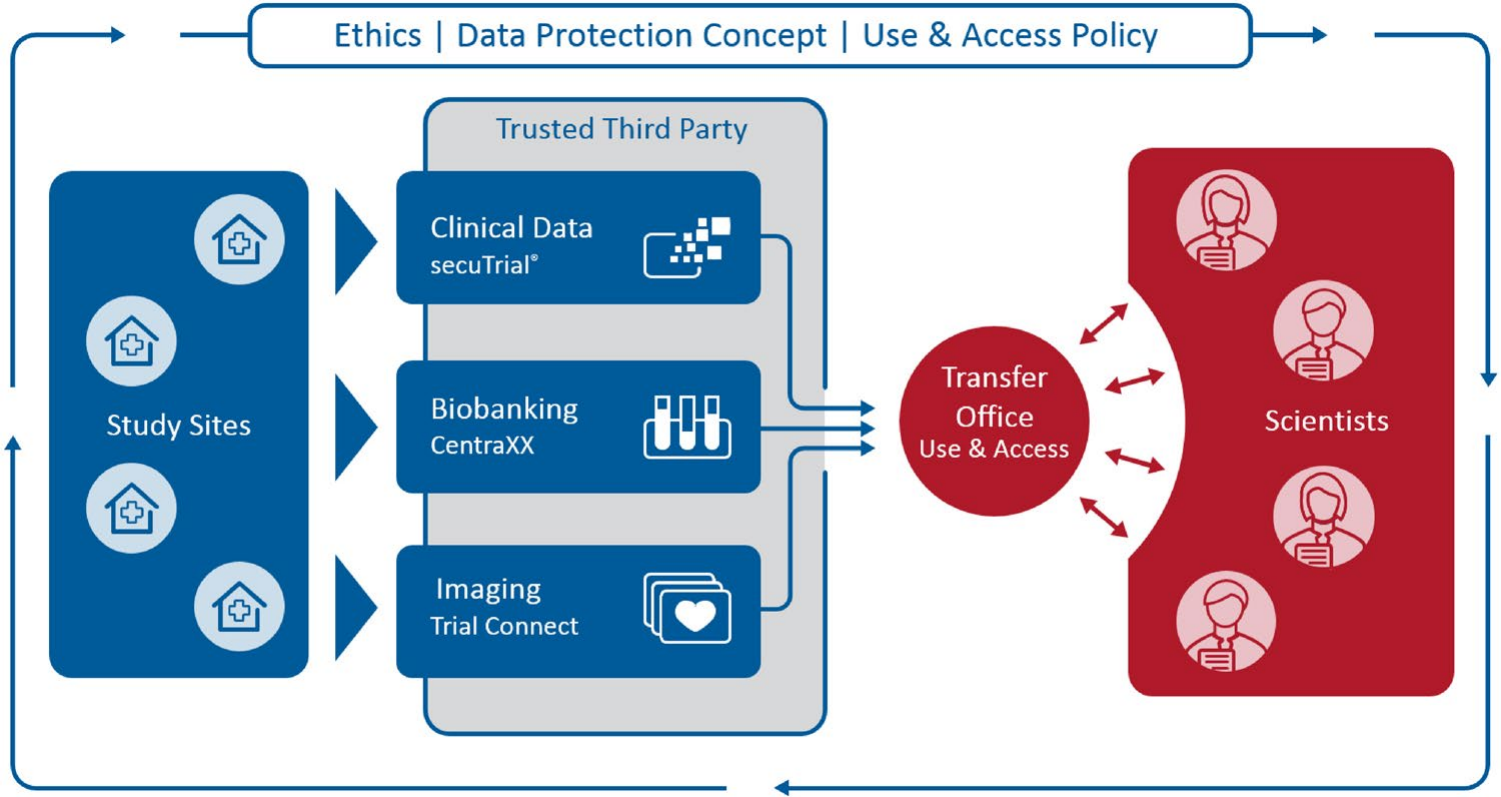
DZHK research platform in the context of data acquisition and biological sample collection from patients recruited in DZHK studies (left, blue) and data as well as biological sample secondary use by scientists (right, red). The framework is defined by the Ethics and Data Protection Concepts as well as the DZHK Use and Access Policy. Study sites primarily use the IT systems for data documentation, while scientists interact with the Transfer Office. The Trusted Third Party enables record linkage and ensures proper pseudonymisation and adherence to informed consents

At the beginning, the DZHK defined needs and processes within working groups. This included ethical and legal aspects and data protection considerations. Furthermore, the definition of study procedures as well as of a modular data set system was concerted to achieve high standardisation across all future DZHK funded studies. While data collection was bound to occur decentralised at all study sites, data storage and handling was decided to be handled in central structures. After definition of elementary processes, discussion about hard- and software solutions suitable for the purposes started. The clear separation of data capture (blue part in Fig. 2) and data analysis (red part in Fig. 2) enabled the DZHK to start recruitment into clinical studies quickly as the main efforts were initially concentrated on data capture.

The establishment of this sustainable structure was a central strategic decision within the DZHK. In 2015, considerable amounts of DZHK budget were assigned to the establishment of the DZHK clinical research platform.

## Principles of the clinical research platform

It is mandatory to adhere to the DZHK use and access policy and to utilise the collaborative research platform for data capture. This is a pre-requisite for funding of clinical studies, i.e., clinical trials, registries and cohorts with a DZHK funding above 50%. This approach has been chosen to avoid troublesome and quality-reducing post-hoc harmonisation of data and results. This new platform sets the basis for a highly standardised and easily accessible data collection (Fig. 2).

Another important agreement of all DZHK partner institutions was to hand over the rights to use data and biological samples collected in DZHK studies to the legal entity DZHK. According to a Public Open Data strategy, scientists worldwide can apply for their use centrally (Fig. 2, red side). After performed quality assurance basis data sets and basis biobanking samples from cohorts, registries and clinical studies are available without any delay. However, for cohorts and registries PIs have a 2-year veto right in case of an application from other scientists. Principal investigators of clinical studies hold the exclusive right for use of data and biological sample for a 2-year embargo period. After the embargo left over material of the study biobanking is transferred to the DZHK Heart Bank and then accessible like the basis biobanking for secondary use. Applications for usage of data and biological samples are discussed in an interdisciplinary Use and Access Committee and the recommendation is given to applicant and Board of Directors.

The overarching objectives of the research platform are.

A. high-quality through standardisation of prospective data and biological sample collections among all DZHK studies
B. Development of sustainable centrally accessible repositories for standardised storage of data and biological samples in compliance with general legal regulations, data protection, and ethical requirements as well as the FAIR principles (**F**indable, **A**ccessible, **I**nteroperable, and **R**e-usable) [5].

Several DZHK sites contribute to the research platform and fulfil important and complementary tasks (Table 1).

**Table 1:** Tasks, responsible DZHK sites and persons

Responsibility	DZHK site	Responsible persons
Central coordination	Berlin	Board of directors, J. Hoffmann, M. Nauck*
Clinical data	Göttingen	D. Krefting, S. Hanß
Biobanking	Greifswald, Berlin	M. Nauck, C. Schaefer, I. Wallrabenstein
Imaging	Berlin, Munich	T. Kühne, J. Schaller, R. Lorbeer
Trusted third party	Greifswald	W. Hoffmann, D. Stahl
Ethics coordination office	Munich	A. Peters, M. Kraus
Transfer office	Göttingen	D. Krefting, S. Hanß

*Spokesman of the Clinical Research Platform of the German Centre for Cardiovascular Research (DZHK)

## A standardised prospective data and biological sample collection

**Fig. 3:**
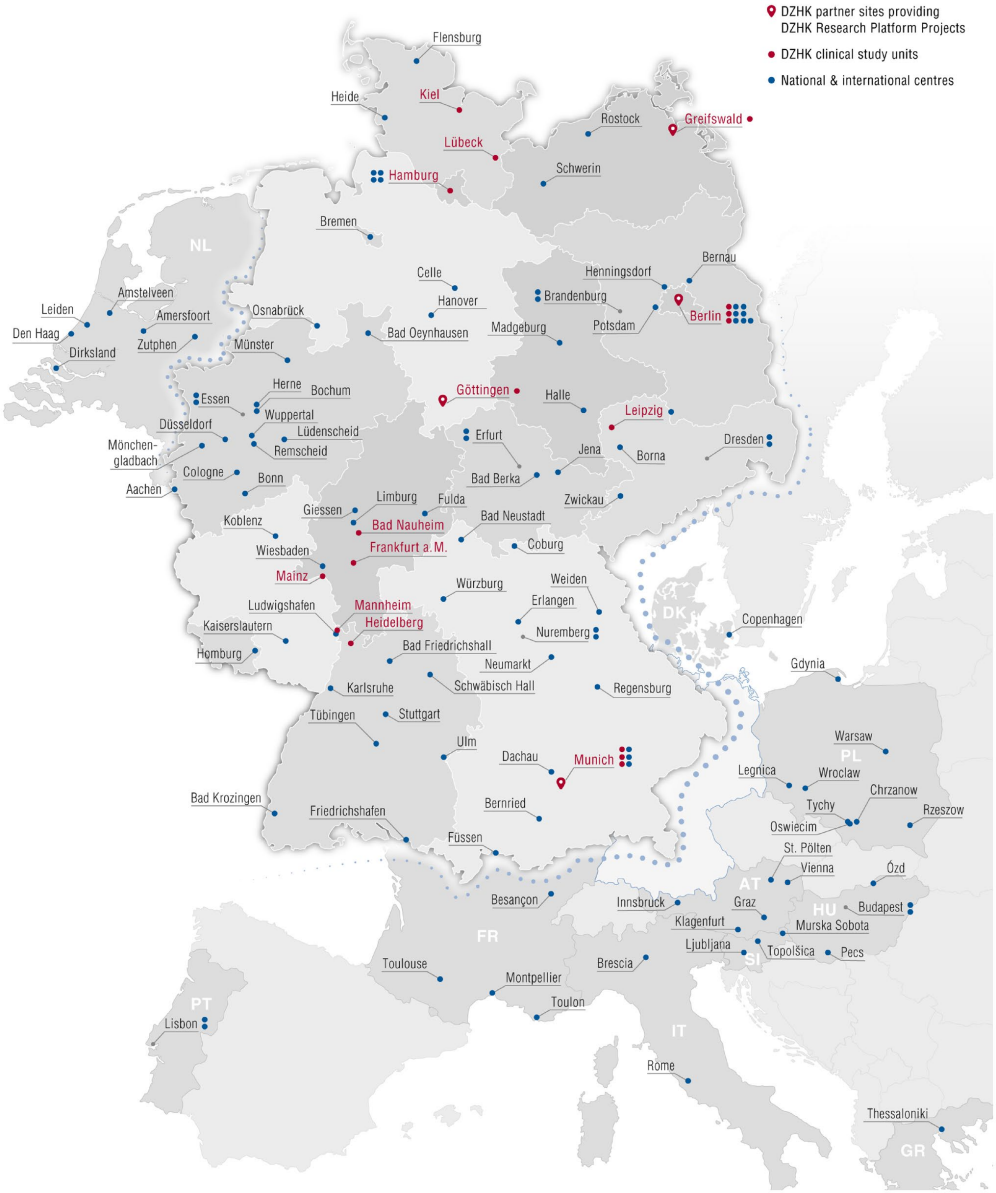
Study sites. Red dots exemplify DZHK clinical study units which are characterised by a close link to the DZHK network and which are, in case of a sufficient recruitment and data collection performance, provided with DZHK clinical staff [3]. Blue dots are external recruitment centres that provide an important role in achieving the high recruitment numbers that are necessary to finish a clinical study within a reasonable time [21]. Red drops stand for DZHK sites providing parts of the clinical research platform

The DZHK is currently funding 27 prospective non-commercial registries, cohorts, and clinical studies (collectively called “DZHK studies”) [6]. Together, these DZHK studies are enrolling participants at more than 100 national and international sites (defined as having recruited at least one participant) (Fig. 3). By December 2022, data of 11,245 patients were contributed to the DZHK data pool.

### DZHK studies: current status

The following five DZHK studies have already been completed.

APPROACH-ACS-AF-DZHK7 is the first trial dedicated to Acute Coronary Syndrome (ACS) patients, testing whether in terms of bleeding a dual antithrombotic therapy (DAT) with new oral anticoagulants (NOAC) is superior to a triple-antithrombotic therapy (TAT) regimen with Vitamin-K antagonists (VKA) in high-risk ACS patients with Atrial Fibrillation (AF). DAT based on NOAC compared to TAT based on VKA reduces bleeding complications, but increases stent thromboses in patients with AF undergoing percutaneous coronary intervention (PCI). The aim of the multicenter prospective, randomized, open-label, blinded endpoint trial APPROACH-ACS-AF-DZHK7 was to investigate, whether a DAT based on TAT-regimen based on VKA can reduce bleeding complications in older patients with AF and ACS undergoing PCI.

The clinical study SMART-MI-DZHK9 was the first to evaluate novel ECG biosignals assessing autonomic function in a prospective randomised way. In a thus identified high-risk post-infarction group with cardiac autonomic dysfunction and only mildly reduced pump function, an implanted heart monitor could better detect those at risk for developing serious and clinically relevant arrhythmic events, when compared to conventional follow-up care.

The prospective, randomised, controlled and multicenter Ex-VAD-DZHK11 trial assessed the effects of a supervised ET program on peakVO2, 6-Minute-Walk-Distance and QoL in patients with implanted LVAD. Whether the results will impact the guidelines and recommendations in the field of end-stage HF will depend on the primary publication, which is currently under consideration.

The HFpEF-stress-DZHK17 trial demonstrated accurate non-invasive detection of HFpEF using exercise stress real-time Cardiovascular Magnetic Resonance (CMR). Reconfirmation in multicenter prospective research studies will be required to establish widespread routine clinical use and guideline incorporation.

PRAISE-DZHK19 I DZNEB001: Troponin elevation is a relevant clinical conundrum in patients with acute ischemic stroke. The PRAISE study clarified the significance of high-sensitivity cardiac troponin elevation with respect to yield of coronary angiography and diagnosis of myocardial infarction in these patients. Guideline has not yet been revised. As the measurement of troponin is recommended in guidelines, it is assumed that the results are guideline relevant.

Table 2 gives an overview of all funded DZHK studies. These information is also available on the DZHK homepage [6].

**Table 2:** Acronym, title, principal investigators (PI), recruitment status, and important publications of DZHK studies

Acronym	Title	PI	Recruitment
TORCH-DZHK1	Translational registry for cardiomyopathies (R) [7, 8]	Prof. Dr. Hugo A. Katus, Prof. Dr. Wolfgang Hoffmann	Completed
TransitionCHF-DZHK2	Systolic dysfunction to congestive heart failure cohort study (C)	Prof. Dr. Gerd Hasenfuß, Prof. Dr. med. Rolf Wachter, Prof. Dr. Frank Edelmann	Ongoing
VAD-DZHK3	Early versus emergency left ventricular assist device implantation in patients awaiting cardiac transplantation (CS)	Prof. Dr. Volkmar Falk, Prof. Dr. Christoph Knosalla, Prof. Dr. Gerd Hasenfuß, Prof. Dr. Tim Friede	Ongoing
TOMAHAWK-DZHK4	Immediate unselected coronary angiography versus delayed triage in survivors of out-of-hospital cardiac arrest without ST-segment elevation (CS) [9–11]	Prof. Dr. Steffen Desch, Prof. Dr. Holger Thiele	
FAIR-HF2-DZHK5	Intravenous iron in patients with systolic heart failure and iron deficiency to improve morbidity and mortality (CS) [10, 11]	Prof. Dr. Dr. Mahir Karakas, Prof. Dr. Stefan Anker	Ongoing
DEDICATE-DZHK6	Randomized Trial of TAVI versus SAVR in Patients with Symptomatic Severe Aortic Valve Stenosis and Intermediate Risk of Mortality (CS) [12]	Prof. Dr. Stefan Blankenberg, PD Dr. Moritz Seiffert	Completed, follow-up continuing
APPROACH-ACS-AF-DZHK7	Apixaban versus Phenprocoumon: oral anticoagulation plus antiplatelet therapy in patients with acute coronary syndrome and atrial fibrillation (CS)	Prof. Dr. med. Reza Wakili, Prof. Dr. Steffen Massberg	Completed
SPIRIT-HF-DZHK8	SPIRonolactone In the Treatment for Heart Failure (CS)	Prof. Dr. Frank Edelmann	Ongoing
SMART-MI-DZHK9	Implantable cardiac monitors in high-risk post-infarction patients with cardiac autonomic dysfunction (CS) [13–16]	Prof. Dr. Stefan Kääb, Prof. Dr. Steffen Massberg	Completed
CAVA-ADHF-DZHK10	Ultrasound evaluation of the inferior vena cava in addition to clinical assessment to guide decongestion in acute decompensated heart failure: a pilot study (CS) [17]	PD Dr. med. Alexander Jobs, Prof. Dr. Holger Thiele	Completed
Ex-VAD-DZHK11	Exercise Training in Patients with Left Ventricular Assist Device (CS) [18]	Prof. Dr. Frank Edelmann, Prof. Dr. Volkmar Falk, Martin Halle	Completed
Decipher HFpEF-DZHK12	Validation of Cardiovascular Magnetic Resonance against Invasive Haemodynamics in Patients with Heart Failure with Preserved Ejection Fraction (CS)	Prof. Dr. Eike Nagel	Ongoing
CTSN-TVR-DZHK14	Evaluating the benefit of concurrent tricuspid valve repair during mitral surgery (CS) [19]	Prof. Dr. Volkmar Falk, Annetine C. Gelijns	Completed, follow-up ongoing
SCREEN AF-DZHK15	Home-Based Screening for Early Detection of Atrial Fibrillation in Primary Care Patients Aged 75 Years and Older (CS) [20]	Prof. Dr. med. Rolf Wachter, David Gladstone, Prof. Jeff Healey	Completed, follow-up ongoing
CLOSURE-AF-DZHK16	Left atrial appendage CLOSURE in patients with Atrial Fibrillation at high risk of stroke and bleeding compared to medical therapy (CS) [21–24]	Prof. Dr. Ulf Landmesser, Prof. Dr. Ingo Eitel, Prof. Dr. med. Leif-Hendrik Boldt	
HFpEF-stress-DZHK17	Cardiovascular magnetic resonance real time exercise stress testing in heart failure with preserved ejection fraction (CS) [25–28]	Prof. Dr. Dr. med. Andreas Schuster	Completed
METRIS-HF-DZHK18	Effect of Metformin in insulin resistant patients with heart failure with reduced ejection fraction (CS)	Prof. Dr. Dr. Wolfram Döhner, Prof. Dr. Tim Friede	Ongoing
PRAISE-DZHK19 I DZNEB001	Prediction of Acute Coronary Syndrome in Acute Ischemic Stroke (CS)	Prof. Dr. Matthias Endres, Prof. Dr. Ulf Landmesser, Prof. Christian Nolte	Completed
BioVAT-HF-DZHK20	Safety and Efficacy of Induced Pluripotent Stem Cell-derived Engineered Human Myocardium as Biological Ventricular Assist Tissue in Terminal Heart Failure (CS)	Prof. Dr. Wolfram-Hubertus Zimmermann, Prof. Dr. Tim Seidler, Dr. Ahmad Fawad Jebran	Ongoing
TORCH-Plus-DZHK21	TranslatiOnal Registry for CardiomyopatHies-Plus (R) [7]	Prof. Dr. Benjamin Meder	Ongoing
EXAMINE-CAD-DZHK22	First prospective randomized trial to examine a differential therapeutic response in symptomatic patients with non-obstructive coronary artery disease after coronary physiological testing (CS)	Prof. Dr. Ulf Landmesser, Prof. Dr. med. Barbara Stähli	Ongoing
CMR-ICD-DZHK23	Cardiac Magnetic Resonance guidance of Implantable Cardioverter Defibrillator implantation in non-ischaemic dilated cardiomyopathy (CS)	Prof. Dr. Ingo Eitel	Ongoing
TRICI-HF-DZHK24	Tricuspid Intervention in Heart Failure (CS)	Prof. Dr. med. Jörg Hausleiter, PD Dr. Thomas Stocker, Prof. Dr. Steffen Massberg	Ongoing
Reduce-MFA-DZHK25	Effect of anti-fibrotic therapy on regression of myocardial fibrosis after transcatheter aortic valve implantation (TAVI) in aortic stenosis patients with high fibrotic burden (CS)	Prof. Dr. Miriam Puls, Prof. Dr. med. Elisabeth Zeisberg	Ongoing
TRINITY-DZHK26	A multicenter, randomized, double-blind, placebo-controlled trial evaluating immunosuppressive treatment in patients with chronic virus-negative inflammatory cardiomyopathy (CS)	PD Dr. med. Ulrich Grabmaier, Prof. Dr. Steffen Massberg	Planned
CABA-HFpEF-DZHK27	Catheter-based ablation of atrial fibrillation vs. conventional treatment in patients with heart failure with preserved ejection fraction (CS)	Dr. med. Abdul-Shokor Parwani	Planned
GECT-DZHK28	A first-in-human feasibility study to evaluate the safety (and short term effectiveness) of the autologous GrOwnValve transcatheter pulmonary heart valve CS)	PD Dr. Boris Schmitt	Planned

*CS* clinical study, *C* cohort, *R* registries

**Fig. 4:**
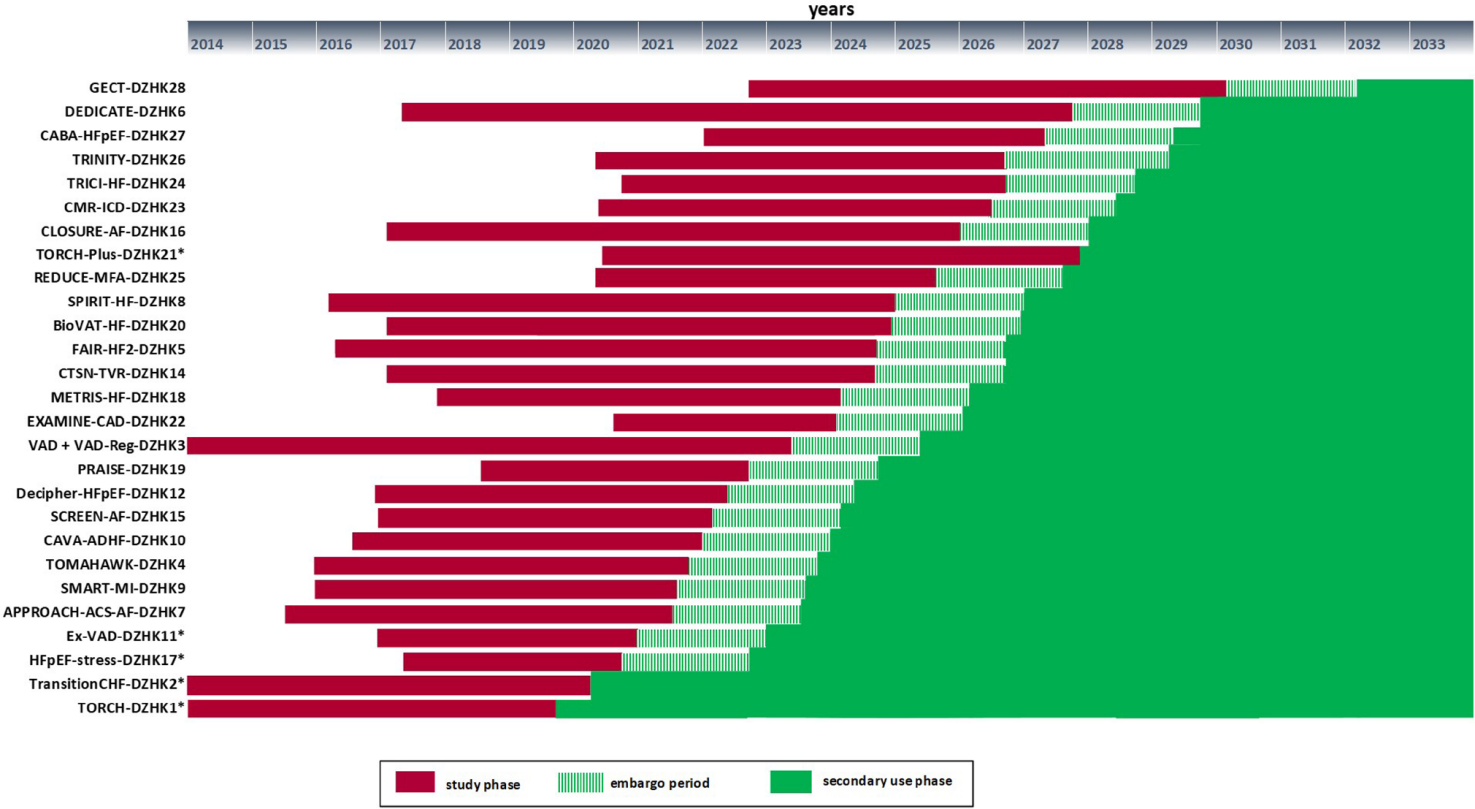
Timeline of DZHK clinical studies with their different phases. The DZHK research platform supports all studies in the trial project phase including recruitment (red). The embargo period (green striped box) follows during which studies conduct their analyses. Typically two years after the end of the study data and biological samples are transferred to the DZHK Heart Bank and are available for secondary use (green). *As of December 2022 embargo period ended

Currently, data sets and biological samples (e.g. serum, EDTA-plasma, citrate-plasma, urine and buffy-coat each) from approx. 7,000 patients are available. Among them 73% are male and 27% are female, the bigger part of them has a cardiomyopathy and also more than the half have cardiac insufficiency. For two clinical studies, the embargo period has ended and scientists around the world can apply for data and biological samples of these studies, one cohort and two registries from the DZHK Heart Bank (Fig. 4).

## Elements of the DZHK research platform

### Common data model

To allow for analyses of clinical data across clinical studies with different disease entities and various treatment and randomisation schemes, the DZHK chose to define a Common Data Model with a mandatory basic data set and an extensive set of optional standardised modules for common cardiological procedures. The basic data set consists of 42 items and focuses on known cardiovascular risk factors, previous diagnoses, and interventions. It was derived from the CodeBook of the Competence Network Heart Failure [29]. Optional modules are available for the assessment of the 6-min walking test (6-MWT), cardiac catheterisation, cardiomyopathy diagnostics, depression, echocardiography, electrocardiogram, laboratory results, medication, MRI, and cardiopulmonary exercise testing (CPET). Each module consists of standard operating procedures (SOP) accompanied by electronic case report files (eCRF) templates and are only used if the study protocol mandates the respective procedure [30]. Optional modules do not have to be used in their entirety. Instead, it is recommended to only use those parts of the module that are relevant for the individual study. This ensures a compact and feasible eCRF and keeps the documentation workload low, while also maintaining standardisation across studies (Table 3). Additional novel study-specific items may be implemented. The Common Data Model including the basic data set and all optional items are available in German and in English. The complete collection (including metadata like units of measure) is published online as part of the DZHK Data Catalogue [30].

**Table 3:** Clinical modules of the DZHK research platform applied in selected DZHK studies current status as of December 2022

Modules	Number of clinical studies
Clinical basis data set	24
Biobanking basic set	24
Anamnesis	24
Laboratory	22
ECG	24
Medication	17
Cardiac catheterisation	8
6-MWT	12
CPET	5
Echocardiography	14
MRI	5
Depression	5

The Common Data Model applies to biological samples as well. Along with the clinical basic data set the collection of a basic biobanking set consisting of liquid samples is mandatory in DZHK studies. In addition, study specific biobanking sets can be collected at different time points, if needed.

A quality management system (QMS) is implemented throughout the DZHK. SOPs describe processes and regulations [31]. It is important to note that in Germany medical facilities are required to implement and maintain a QMS according to the national Code of Social law, book V (SGB V). Therefore, the DZHK had to develop a QMS that fits all clinical QMS from DZHK sites and partners and still fulfils the purpose of standardisation without jeopardising ease of use. To achieve this, the essential parts of SOPs are highlighted to tell them apart from those sections that can be adapted to the local QMS. Even adopting only those parts of DZHK SOPs, which are marked as essential, is possible. This offers the advantage of keeping the documents of a QMS within a given DZHK site in a familiar design and avoid conflicting SOPs.

### Complex research procedures: concept of quality levels

It is mandatory for each DZHK study and partner institution to adhere to established DZHK standards. This ensures a high level of data quality and superior comparability (see Table 4, level 1). All relevant processes in the DZHK are defined in SOPs.

**Table 4:** Overview over DZHK quality levels

	DZHK quality levels
Implementation	
Level 1	The examination is performed in accordance with the requirements laid down in clinical guidelines (state-of-the-art), which is documented as level 1 in DZHK SOPs
Level 2	The examination is performed in accordance with more sophisticated requirements above state-of-the-art. The minimum requirements for ensuring the quality of the implementation and of the examiner are defined in DZHK level 2 SOPs
Level 3	The examination is performed in accordance with detailed requirements including certification of the examiner: determination of intra- and inter-observer variability (standard of epidemiological studies) as defined in DZHK level 3 SOPs

In some cases an even higher quality level is required. For example, if the standard measurement or investigation procedures are not able to carve out differences in the study population, more sophisticated measurement or observation procedures are necessary. For this purpose, additional quality requirements are defined as level 2. If even higher standards are required, the DZHK allows for level 3, which represents the highest level of quality. In this case, investigators need to be certified and the measurement uncertainty of measurement procedures needs to be described and documented.

## Sustainable data and biological sample repositories

In the DZHK, data are stored and managed centrally whereas biological samples are physically stored directly at the sites that collected them. Designated partner sites are responsible for collection of clinical data (CDMS, Göttingen), meta-data on biological samples (LIMS, Greifswald/Berlin), and imaging data (IDMS, Berlin). CDMS, LIMS, and IDMS are based on mature commercial systems that have been configured or customised for the needs of the DZHK.

### Clinical data management system

Clinical data are collected centrally through electronic Case Report Forms (eCRF) in the web based clinical data management system (CDMS) secuTrial (Gesellschaft für interactive Medien mbH, Berlin, Germany) which is hosted at the University Medicine Göttingen. Since secuTrial is a validated software according to FDA 21 CFR part 11 it is suitable for all kinds of clinical studies including drug and device trials. To increase the data quality, automated plausibility checks have been implemented that assess data input in the eCRFs with direct feedback to the user. These plausibility checks are harmonised like the common data model itself and are valid over all DZHK studies. Additionally, the CDMS provides audit trails and data quality reports.

### Decentralised biobanking and central metadata documentation

The biological samples collected within the DZHK platform are stored locally at the collecting sites. An important feature though is that the documentation of storage place and meta-data of biological samples is done in a centrally operated laboratory information system (LIMS, CentraXX (KAIROS GmbH, Bochum, Germany), hosted in Greifswald) that is accessed by the sites through a web application. The combination of decentral biological sample processing and storage and central metadata storage is beneficial for efficient scientific use of these resources. To further improve the performance of the research platform centralisation of basis biobanking sets will be implemented at two sites in 2024.

According to the modular structure of data collection also biological samples were grouped into a mandatory basic biobanking set for all DZHK studies (Table 5) and optional study specific biobanking sets.

**Table 5:** DZHK basic biobanking set

Biological sample	Aliquot volume	Aliquot quantity
Serum	300 µl	10
EDTA plasma	300 µl	10
Citrate plasma	300 µl	4
Urine	300 µl	8
Buffy coat	< 300 µl	2

Provided the participant’s consent, all DZHK studies are required to collect the basic biobanking set at the baseline visit prior to any intervention. The purpose of the DZHK basic set is to build a large resource of high quality, well documented, and standardised biological samples with broad availability. Therefore, establishing standardised and traceable processes for biological sample handling represented a major challenge. In 2011, Baker and Simeon-Dubach et al. demonstrated the pitfalls of lacking information on sample quality and the danger of drawing wrong conclusions [32, 33]. To assure standardisation and safe sample identification the DZHK decided to use aliquot tubes with unique 2-D codes and a sample volume of 300 µl. At the partner sites, freezing equipment at – 80 ℃ varies from stand-alone freezers in remote areas to fully automated large-scale biobanks in established medical research facilities.

The DZHK LIMS was customised based on the commercially available CentraXX system. It is a process-based system that allows the user to move to the next step only if the previous one is finished. The sample processing is steered by workflows, which are defined in the biobanking LIMS. The user is led through the LIMS by these workflows, resulting in a real-time documentation of each quality relevant step, avoiding errors by skipping or delayed documentation in the final IT system. The LIMS assists the study nurse for each individual patient to be included in a given study, which increases data quality. Study-specific processes and SOPs are integrated into LIMS processes. On inclusion of a patient the LIMS will print barcodes for the labelling of primary biological sample vials, e.g., for blood collection, that are needed in this specific visit according to the study protocol. When primary tubes are processed, the web-based LIMS follows these steps and offers an eCRF in which processing type, e.g., centrifugation and conditions, as well as processing times are documented at the very moment they occur in the work flow. Aliquoting is also documented in the LIMS with data input either manually or through pipetting robots and their interface. Storage of samples in freezing devices is also documented by scanning the unique 2-D code. At the end of the processes, the aliquots have to be stored at − 80℃ locally. Further on, the LIMS offers audit trails and strongly facilitates quality assurance measures.

In the first phase of the DZHK, when CentraXX had not been implemented, prelabelled biobanking sets were sent to the clinical study units. In these cases the processing data was documented in SecuTrial. This procedure is still valid in small recruitment centres outside the DZHK.

### Image data management system

Imaging data can be uploaded and managed in a centralised image data management system (IDMS). IDMS was established to provide storage services for clinical studies and to make imaging data available for post-processing and reuse. Required information from the CDMS—subjects, visits, and some clinical data—is synchronised to link imaging and clinical data to the visit context. The raw data are stored in the DICOM standard. Since 12-channel resting ECG-signals can also be converted to DICOM format without information loss, the IDMS is used to store biosignals as well. The IDMS is a customised version of TrialComplete by Deutsche Telekom Healthcare Systems GmbH (Bonn, Germany). It operates redundantly in data centres in Almere and Aalmeer (The Netherlands). Primary focus of this technical platform is to provide a low technical barrier for a quick start of data uploading in clinical trials. For high data standardisation, working groups defined data acquisition SOPs for 12-channel resting ECG, transthoracic echocardiogram and cardiac magnetic resonance imaging. In studies with a certain modality the imaging protocol is based on the standardised DZHK protocol and further adapted to the study needs. Central image analysing units (CoreLab) of the studies are able to check and analyse the data quality by image viewer or image download. Results of CoreLab analyses can be uploaded and entered in IDMS eCRFs.

### Compliance with legal regulations, data protection, ethical requirements, and FAIR principles

Sophisticated ethics and data protection concepts secure the work of DZHK researchers. Several working groups were engaged in the development and had to align their work prior to implementation. Moreover, the FAIR principles were considered as being indispensable for the DZHK. All objectives are implemented while setting forth highest standards regarding ethics and privacy protection. The goals are put into effect within a framework consisting of the Ethics Concept, the DZHK Use and Access Policy, and the Data Protection Concept [34]. This framework is the foundation of a sustained and broad re-usability of data and biological sample collected within DZHK studies.

Clinical research requires participants who make an informed decision to participate in a study. During this process, they grant the DZHK usage rights to their data and ownership of their biological sample—without a fixed time limit but limited to improvement of prevention, detection and treatment of diseases. An important task of the DZHK is to ensure the privacy of participants and the security of its collected data, while also promoting adequate and—in the case of limited quantity of biological samples—efficient use of data and samples. Moreover, all of this has to be performed in a transparent and comprehensible manner. Study participants, their relatives, and other interested parties are able to gain information about DZHK studies in general via a special patient information platform (PIP). In addition, the PIP provides information on the secondary use of data and biospecimens from the DZHK Heart Bank [35].

### Ethics concept

In the DZHK, a uniform ethics concept regulates what may be done with data and samples, how patients are informed about data management and data use and what possibilities they have to withdraw their consent. It was developed by the Ethics Coordination Office in Munich together with partners of the research platform. In accordance with its philosophy, the DZHK enables interdisciplinary and multiple use of data and biological samples by scientists inside and outside the DZHK. The Ethics Coordination Office in particular supports DZHK scientists to efficiently implement high quality research in the DZHK study pipeline and with DZHK resources from an ethics perspective. The ethics concept outlines the structural and ethical principles of the DZHK research platform, describes the concept of informational separation of powers in data management, and is applicable to different types of studies—ranging from registries to randomised controlled studies.

The DZHK ethics concept is based on a modular approach for a broad consent [36–38]. Documents for participant information and consent are carefully aligned across all modules. Basic consent modules for processing data and biological samples are mandatory for every clinical DZHK study. These modules are supplemented with additional study-specific modules based on the needs of each clinical study. This method ensures that for a certain use case, e.g., an application to use biological samples for genetic analyses, the necessary informed consent verification can be performed electronically for all participants across all studies.

The ethics concept is closely linked to all other processes of the DZHK. In view of this background, the modular approach enables the DZHK to achieve a high degree of standardisation across all studies without jeopardising flexibility of the individual studies.

All patient information and consent forms for the widely differing multicentre clinical studies have been presented to and were accepted by over 50 German ethics committees. In some cases, questions from the leading ethics committees had to be answered before a final positive vote. In about 25% of the submissions to the ethics committees of participating study centres, individual parts had to be discussed and additional clarifications had to be provided. However, every single study centre was able to obtain a positive vote from their competent ethics committee.

Because of the involvement of various ethic committees, more than 200 consent templates modules were implemented in the Independent Trusted Third Party (TTP) for the several projects. From an ethics committees' perspective biomaterial collection should be recognisable as a separate aspect of consent or require a separate consent form. Therefore, 187 templates for the general study, partly including separate modules for biobanking, 66 specific templates for biological sample collection as well as 16 templates for sub studies or other purposes (as of December 2022) were implemented.

So far 244 consent withdrawals (approx. 1.1% of all consented DZHK participants), 566 study exclusions (approx. 2.5% of all consented DZHK participants) and 247 limitations to re-contact (approx. 1.1% of all consented DZHK participants) had to be processed within the DZHK research platform as of December 2022.

### Data protection

**Fig. 5:**
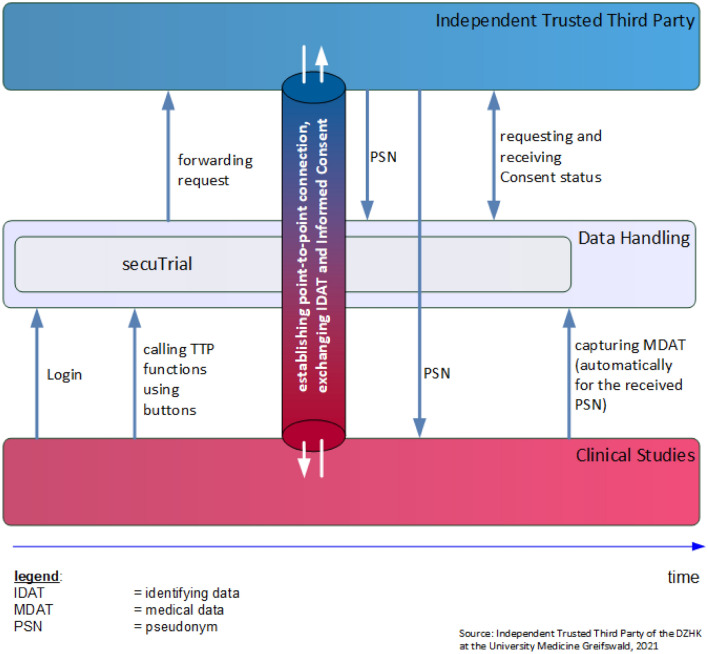
Seamless integration of TTP-system into the clinical data system incl. interaction of the involved entities

The data protection concept was developed to ensure that the participants’ rights are respected and that the complex infrastructure complies with all regulations, most importantly the EU General Data Protection Regulation (EU-GDPR) [39]. It is based on the guideline regarding data protection in medical research projects (Version 2.0) published by the TMF, which is well established in Germany [40]. A key aspect of this guideline is the informational separation of powers, which is achieved by separating medical and identifying data as well as using different identifiers for participants in all individual documentation systems. A merging of data from different systems can only be achieved by involving the TTP to map the individual identifiers to a single identity. Upon inclusion patient codes (pseudonyms) are issued. This is achieved by connecting the clinical data system (secuTrial) to the system of the TTP. Due to the deep seamlessly integration of the TTP-system into secuTrial, the user does not need to change systems, but instead can continue working in the clinical data entry system (Fig. 5). The integrated direct point-to-point use of TTP software ensures a fast and smooth data entry process without waiting time for neither the patient nor the DZHK staff. Person-identifying data are stored only within the TTP, whereas all other DZHK systems have only system specific codes (pseudonyms) so that no conclusions can be drawn about individuals. After the initial registration of a patient, each DZHK system only operates with their independent codes (pseudonyms). The TTP software solutions of the University Medicine Greifswald are in-house developments [41]. The TTP-system [38], like all DZHK-IT-systems, is web-based and the basic functionality does not require any installation of hardware in the individual study centres (zero footprint), which is a key feature for easy enrolment of new study sites. However, the TTP requires additional client certificates as data protection measure for transferring sensitive person-identifying data. Furthermore, all systems require authentication by username and password, support fine granular rights and roles management, and all connections are encrypted using state-of-the-art encryption (at least TLS 1.2).

### Use and access policy

The policy governs the transfer of data and biological samples to scientists and is the responsibility of the Use & Access Committee. The Use and Access Policy was reconciled, approved by the legal departments of all 28 DZHK member institutions, and enacted by the DZHK in 2014 (amended in 2021) [34]. According to the individual DZHK studies’ endowment contracts, all data and biological samples collected by DZHK studies are subject to this Use and Access Policy. The central aspect of this policy is that the DZHK association is owner of the collected biological sample and has—in compliance with consents and withdrawals—usage rights to all consented data collected in DZHK studies. Of course, this is also incorporated in the informed consent templates and represents an important part of the Ethics Concept. This transfer of rights enables the DZHK to make strategic decisions regarding the secondary use of its data and biological samples.

There are two intended cases for data and/or biological sample utilisation: 1. Principal investigators should notify the Use and Access Committee if they want to use data and/or biological samples for their own study in case of further research questions that go beyond the actual study question. 2. If researchers desire data and/or biological samples across the DZHK Heart Bank, an application is required [42].

Researchers inside and outside the DZHK can verify availability of data and biological samples through a Feasibility Explorer prior to applying [43]. The Use and Access Committee evaluates notifications and applications with respect to the scientific approach as well as—in a cursory manner—ethical and legal standards. Criteria for the evaluation of the Use and Access Committee are integrity and scientific reputation of the applicant, scientific concept of the investigation planned, including number of cases and analytical strategy, consistency of the proposal with the goals and regulations of the DZHK, and feasibility in view of available resources and scientific aspects. Additionally, an ethics vote for the specific project is required for usage applications. The final recommendation of the Use and Access Committee is given to the applicant and the Board of Directors will subsequently be informed. Data and biological samples for positively recommended secondary use projects are made available to the applicant via the Transfer Office, which integrates clinical data, biological samples as well as image and omics data.

## Quality assurance

The quality assurance process of informed consent documents implemented by the TTP showed that manually entered information of paper-based IC forms into an electronic form is error-prone. Overall, only 80.4% (as of December 2022) of ICs are initially entered correctly from paper-based version into the database. Therefore, a comprehensive quality assurance is conducted by the TTP to correct errors.

Quality assurance for the delivery of biological sample has also been implemented. To provide high-quality biological sample and related data and to ensure smooth use and access processes along with an easy release and delivery an internal quality control delivery study was conducted in 2018. A usage application was simulated including various DZHK clinical project units and overall 100 biological samples from DZHK Heart Bank. Briefly, the application procedure and the delivery of biological samples to the recipient was in time, the laboratory analysis proved a high quality of biological samples comparable to the quality of German National Cohort (GNC) samples [44].

To close the quality loop, internal DZHK audits are performed at DZHK clinical study units. Results from these are reported and discussed centrally in the meeting of members to achieve optimisation.

## Using the DZHK research platform

### How to get access to data and biological samples

According to the DZHK Use and Access Policy temporary, appropriate, non-exclusive, and non-transferable rights of use may be granted to an applicant under the condition that data and biological sample are used for purposes consistent with the objectives of the DZHK and will not compromise the interests of DZHK. Commercial exploitation is excluded while well-considered industrial cooperation projects are intended. The secondary use of data and biological sample from the DZHK Heart Bank is orchestrated and supported technically by the Transfer Office and procedurally and formally by the Use and Access Office. Both act as an interface between DZHK Heart Bank and scientific community [45].

### Selection of data and biological sample

The DZHK offers two public tools to scientists planning a research project with data and/or biological samples from the DZHK Heart Bank: (1) The Data Catalogue, which is a formal description of all DZHK wide standardised data items. (2) The Feasibility Explorer [43]. The Feasibility Explorer is a tool developed for the purpose of interactively exploring available clinical data, imaging data, and biological sample by filtering for specific parameters, and specifying collectives to be used for application process. According to the selected parameters the applicant sends a usage application to the Use and Access Office. After a formal application check the Use and Access Office forwards the application to the Transfer Office for a first availability check. The review process of the Use and Access Committee is started by forwarding the first availability statement together with the application by the Use and Access Office. Within 4 weeks, the Use and Access Committee gives a recommendation. In case of a positive recommendation, a transfer agreement is concluded between the DZHK and the applicant’s institution. The data and biological sample retrieval are then initiated by the Transfer Office.

### Delivery of data and biological samples

Once both parties have signed the transfer agreement, the Transfer Office initiates data and biological sample delivery process.

For clinical data a final consent check by TTP integration is done and clinical data are compiled, stripped of primary identifiers and labelled with export-specific identifiers. The Transfer Office keeps the mapping between original identifiers and export identifiers, ensuring that a connection to the original participant/data set can be made, if necessary. However, the recipient is unaware of this mapping. The compiled data set is then delivered to the recipient via an encrypted file transfer service [43]. The Transfer Office archives an encrypted copy of the delivered data set for replicability purposes.

For biological samples, the Transfer Office instructs the responsible study sites or biobanks to deliver the biological samples directly to the recipient. The instructions contain the list of primary identifiers material type and amount, and export identifiers to be used, ensuring that the recipient can make a connection between received data and corresponding biological sample. The actual transport (e.g. delivery date, choice of courier service, transport conditions) is negotiated directly between study site/biobank and recipient. However, the DZHK provides a biological sample release and shipment SOP for study sites and biobanks and an information sheet for the applicant.

## Lessons learned

At the beginning, acceptance for such a large project had to be raised within the DZHK. The DZHK infrastructure was constructed by scientists guided by and focused on the needs of scientists, facilitating its accurate fit-for-purpose and consequently its wide acceptance.

The DZHK research platform has demonstrated to be a robust, scalable, and flexible research data infrastructure for effective collaboration and for multiple application options. It improves clinical translation of new knowledge and provides established processes for nationwide clinical trials and registry studies. Therefore, the DZHK research platform can handle the operation of many heterogeneous clinical studies in parallel. It provides excellent privacy for the involved participants and easy access to collected data and biological samples for the research community according to the FAIR principles.

After a learning phase, the implementation time from project start to first-patient-in could be brought down to an average of six months with some variation founded in the complexity in regard to content and regulatory affairs of the study or trial. This was possible due to critical reflexion of introduced processes leading to more efficient ones.

The strategy to develop the practical DZHK platform used a “thinking from goal to start” approach and is geared towards the needs of scientists conducting clinical research. In this regard, the DZHK solution comprises many important features. These include, amongst others, the seamless integration of many specialised systems and at the same time ensuring high user-friendliness by single sign-on or innovation solutions to patient rights while ensuring overall performance at the same time.

The DZHK operates as one legal entity (e.V.) holding all rights of data and biological sample usage, and established centrally available data collections, accompanied by protected time intervals for the PIs and the usage of the study specific biomaterial collections. It also clearly defines and practices project-independent coordination between cooperation partners of the structure and other institutions.

The value of data collections is determined by data quality. To ensure high quality data, audits are mandatory for every DZHK study, e.g. on-site monitoring with spot checks of data (source data verification) [8]. Additionally, PIs and study coordinators review data of their respective study site and correct quality issues locally. After the introduction of the infrastructure, it was relevant to continuously verify high quality of data and biological samples processes across all DZHK studies and sites. Therefore, regularly audits/monitoring were established centrally and conducted on site, even virtually during the corona pandemic. Quality management supports improvement of processes and consequently the quality of the DZHK data collection including the quality of handling and storing of biological samples. Quality of biological samples is greatly determined by the preanalytical process, e.g., the collection and preparation until storage. These process steps are monitored tightly by the DZHK research platform through workflows, which result in a process control and live documentation while collecting and preparing the sample. Additionally, depending on the measurands, state-of-the-art quality assurance processes are adhered to upon analysis.

The DZHK platform’s main feature is its modular implementation. It combines different IT-systems to ensure professional documentation of different data types and sources, and enables flexibility. For example, changing regulations like the introduction of the EU-GDPR [39] required adaptations to the Data Protection and the Ethics Concept. However, due to the modular design of the platform and its processes, these changes only required updates in individual components and not a total redesign.

The Central Ethics Coordination Office, which coordinates the standardisation and harmonisation of consent documents, is established as a competent and trustful partner cooperating with study coordination offices and local ethics committees. Main parts of the DZHK research concept are participant information and consent documents to ensure EU-GDPR-compliant research. Local ethics committees often require local adaptations to ICs as prerequisite for a positive vote complicating the harmonisation of IC documents for research—especially, with many different multi-site studies. As a result, individualised IC documents need to be created. To ensure that the key issues of the research platform always remain represented and avoid defibration of the contents of those documents, the DZHK approach proved suitable: a) document finalisation is accompanied by a Central Ethics Coordination, and b) the TTP solution allows for mapping all individualised documents’ parts to pre-defined standardised policies and, thus, mitigates the diversification.

Use and access procedures are implemented and data as well as biological samples can be provided to researchers worldwide. To achieve a high transparency, all process steps are well defined and documented.

To achieve a high quality of biological samples local processing is mandatory and was implemented within the DZHK from the beginning. To support the in-future increasing number of biological samples delivery, the Research Coordinating Committee decided to centralise basis biobanking sets at two sites that will store about 50% each. This process will increase the performance of DZHK Heart Bank and will be finalised until the end of 2024.

Since its foundation, the DZHK platform has evolved to an innovative infrastructure that can serve as a blueprint for other large scale-biomedical projects looking for solutions to collect as well as share data and biological sample. Consequently, when the COVID pandemic broke out, main elements were used as a template to quickly establish the research platform of the University Medicine Network (NUM) [46]. Another example is the German Medical Informatics Initiative (MI-I) which uses established methods and the experience of the research field highlighting the applicability of DZHK’s approach. The MI-I consortium HiGHmed uses the DZHK standard data set. Additionally, DZHK expertise adds value to other large publicly funded initiatives such as other DZG.

The DZHK platform was able to successfully establish itself in the knowledge landscape by closing gaps especially in view of multicentre clinical research.

## Conclusion

In summary, the DZHK has created an innovative, nationally and internationally visible workflow-based research platform for collecting and storing biological samples and highly standardised data in multi-centre research building, one of the few public data sources for prospective health research. Due to its modular structure, the created solution also provides data that are of direct value, not only for the studies for which they were collected, but also for their secondary use. With the beginning of the artificial intelligence era, it is expected that the use of these data will increase in the future.

So far, 27 DZHK studies recruited well over 11,200 participants suffering from major cardiovascular disorders such as myocardial infarction or heart failure. The DZHK collection represents a large—and what is more—very comprehensive and highly standardized data and biosample collection of patients with cardiovascular diseases. In addition to results from the primary studies, the DZHK infrastructure enables a multitude of secondary research projects, increasing benefits for patient care in the future even further.

## Data Availability

Research data are available after formal application. Information can be found here: https://dzhk.de/en/research/data-and-sample-collections/how-to-apply/ [45].

## Declarations

### Employment

Julia Hoffmann, Katharina Eulenburg and Stephanie Lesser are employees of the DZHK. Joachim Krebser was Managing Director of DZHK until Dec 31 2020.
